# Do Compressed Work Schedules Compromise Care Quality? A Five-Year Case Control Study in the Norwegian Long-Term Care Sector

**DOI:** 10.1177/11786329261471203

**Published:** 2026-08-18

**Authors:** Vilde Hoff Bernstrøm, Mari Ingelsrud, Anne-Marthe Rustad Indregard, Karoline Seglem

**Affiliations:** 1 Work Research Institute, Oslo, Norway, OsloMet; 2 Department of Nursing and Health Promotion, Oslo, Norway, OsloMet

**Keywords:** quality of care, adverse events, extended days, compressed work week

## Abstract

**Background:**

In a compressed work schedule, employees work extended shifts (≥10 hours) in exchange for fewer workdays. This shift schedule has been argued to both improve and compromise the quality of care. However, existing research remains limited and inconclusive.

**Objective:**

To assess whether extended shifts within compressed work schedules impact the risk of adverse outcomes among care recipients.

**Design:**

Quasi-experimental case control study.

**Method:**

We combined a nationwide organizational survey from 1,072 long-term care institution in Norway with registry data on service users from 2018-2022. At baseline, all participating organizations operated either a two-shift rotation (day/evening) with fixed night workers or a three-shift rotation (day/evening/night). The intervention group comprised organizations (n=320) that introduced a compressed schedule during the study period; the control group retained their existing shift rotations. Using difference in difference with multiple treatment times we analysed changes in proportion of service users within each organization experiencing at least one adverse outcome per month. Adverse outcomes were identified via diagnostic codes from the Norwegian Registry for Primary Health Care, including infections, chronic ulcers, accidents, and depression.

**Results:**

Overall, we found no difference in the risk of adverse outcomes (b −0.46, 95% CI −0.97 to 0.05). Stratified analyses indicated a significant reduction in adverse outcomes in nursing homes (b −0.79, 95% CI −1.48 to −0.10). However, we found no significant change in residential care (b 0.20, 95% CI −1.11 to 0.70) or home care (b −0.42, 95% CI −1.20 to 0.36).

**Conclusion:**

The findings neither discourage the use of compressed work schedules due to adverse outcomes nor indicate broad improvements. However, the results suggest potential context-specific benefits that warrant further investigation.

## Introduction

The use of extended shifts in healthcare has raised international awareness, as researchers and practitioners debate their impact on employee well-being and the quality of care. Several studies have linked extended shifts to negative patient outcomes, including a higher risk of adverse events.^1-5^ However, most prior research has not distinguished between extended shifts within compressed schedules and extended shifts that increase total weekly hours.^3^ Consequently, adverse outcomes attributed to shift length may result from the combined strain of long shifts and excessive total work hours.^3^

In a compressed work schedule, employees work extended shifts (≥10 hours) compensated by fewer workdays so that total weekly hours remain unchanged. Compressed work schedules are implemented for various reasons from rationalizing staffing resources to improving staff’s work-life balance. Compressed schedules may affect care quality through competing mechanisms. Longer shifts can cause fatigue, impair cognitive function, impair employee performance and increase the risk of errors.^2^ Conversely, fewer shift handovers may reduce communication failures, a well-documented source of adverse events in healthcare.^6^ Compressed schedules may also improve continuity of care, allowing staff to attend to the same patients for longer periods within and across consecutive days, benefiting both users and staff.^7^ Yet, extended periods away from work may leave employees less attuned to changes in patient conditions when they return.^7^ Understanding the net effect of these opposing mechanisms is essential for evaluating the implications of compressed schedules on care quality.

Evidence on the effects of compressed work schedules on performance and organizational outcomes remains limited and inconclusive, and even more so for healthcare settings.^3,8,9^ In a 2008 review, Bambra, Whitehead, Sowden, Akers and Petticrew^8^ identified only four studies specifically investigating outcomes in healthcare settings: two found no or limited differences in errors or performance, while two reported benefits in self-reported quality and continuity of care.^10,11^ In a 2016 review, Dall'Ora, Ball, Recio-Saucedo and Griffiths^3^ included only one study within healthcare, which found lower self-reported quality of care when compressed schedules were combined with rotating shifts.^12^ More recently, Bernstrøm, Alves, Houkes, Lillebråten and Nilsen^9^ identified four studies in healthcare: two reported benefits in self-reported risk of error and safety culture,^13,14^ while two found no significant differences on objective measures.^15,16^ Across all three reviews, the studies were limited to hospital and often acute care settings, most with small samples and short follow-up periods. Overall, there is a need for comprehensive studies assessing the consequences of compressed schedules for care quality in large samples, over longer periods, and using objective measures. There is also a clear gap in knowledge regarding the association between compressed work schedules and service users’ outcomes in non-hospital settings, such as nursing homes and home care. The objective of this study is to examine whether extended shifts within compressed schedules affect the risk of adverse outcomes among care recipients in long-term care settings, and to determine the direction of any such effects.

## Method

### The Intervention: A Compressed Work Schedule

In recent years there has been a stark increase in compressed schedules within Norwegian long-term care institutions, providing a unique opportunity to investigate their consequences.^17^ Using extended shifts as part of the planned shift rotation is only permitted in Norway when it is organized as a compressed schedule. Weekly work hours vary between 34 and 36 hours a week on average for full-time employees in shift work.^18-20^ Most institutions have implemented a compressed work schedule with a mix of extended and shorter shifts. The majority implement a schedule with extended shifts only on weekends and shorter shifts on weekdays, to address weekend staffing challenges.^21,22^ Only a minority of organizations using extended shifts throughout the week have employees exclusively working extended shifts. The clear majority of compressed work schedules have shifts between 12 and 12 ½ hours, although longer shifts occur. Working extended shifts is required to be voluntary. Consequently, organizations implementing a compressed work schedule also have employees working two-shift rotation (day/evening) with fixed night workers or a three-shift rotation (day/evening/night).”

### Study Design

The study combines Norwegian administrative data from 2018 to 2022 and an organizational survey mapping shift solutions in Norwegian municipal health care. In Norway, responsibility for long-term care lies with the municipalities.

In collaboration with the Frisch Centre^23^ we sent a survey to organizations within the municipal health and social sector (i.e. long-term care). We identified relevant organizations in the public registry containing the name, organizational identifier, Industrial code (NACE) and municipality of all Norwegian organizations. We included all Norwegian nursing homes (907 organizations), home care services (1,017 organization), and residential care organizations (2,254 organizations, including shared housing for the elderly and disabled with staff, care institutions for substance abuse, and shared housing for the mentally disabled), in total 4,178 organizations across 357 municipalities. Most organizations are owned by the municipality (n=3,793), while 9% are privately owned (n=388).

We distributed the survey electronically to the municipalities in May 2022, with instructions that the questionnaire should be completed by each organization’s manager or person knowledgeable about the organization’s shift arrangement.

In total, 1,443 organizations responded to the questionnaire, yielding a response rate of 35%. The response rate was highest among the largest organizations (>60 employees) and for organizations within the smallest municipalities. Due to the method of distribution, the response rate was also substantially higher in public organizations (38% in public vs 1% in private).^23^ For a detailed description of the sample see appendix A.

Each organization in Norway has a unique organizational identifier. That identifier was used to combine the survey with administrative data from the Norwegian Registry for Primary Health Care (NRPHC) for all registered service users (e.g. patients) of long-term care providers.

### The Organizational Survey of Shift Solutions

The organizational survey asked each respondent to provide information on their organization’s shift schedule, including if they had each of the following shift schedules per 1 May each year from 2018 to 2022: two-shift rotations with day and evening; three- shift rotations with day, evening and night; extended shifts; and 24-hour shifts. The survey defined extended shifts as: “*Shifts longer than 10 hours that are part of the employees’ rotating schedule, where employees have longer but fewer shifts. Extended shifts can be used throughout the week or only on weekends. We do not refer to long work periods that occur due to special needs or when employees occasionally work double shifts. We also do not mean 24-hour shifts (see below)*”.

Organizations reporting extended shifts were then asked “*On which days of the week did employees work extended shifts during a workweek? 1. weekdays and weekends, 2. only on weekends, 3. Other*”. Based on the organization’s response for the implementation year, they were either categorized as a compressed work schedule (CWS) all week (responded option 1), CWS weekends (responded option 2, and not 1), or other/missing (responded only option 3 or missing data). Organizations in the third category were not included in analyses distinguishing between types of compressed schedule. Organizations that changed type of compressed schedule were censored from the time of change onward.

For complete wording of the questions see appendix B.

### Norwegian Registry for Primary Health Care (NRPHC) Including Data on Adverse Outcomes and Service Users

The Norwegian Registry for Primary Health Care (NRPHC) is an administrative system for health and social care organized within the municipalities. The registry contains data recorded for each individual service user, compiled from two sources: (1) health and social care services provided by the municipality (i.e. long-term care), and (2) primary health-care practitioners. Data from the first source, (1), includes data on the service provided by the municipality, the organization providing the service and on the individual user receiving the services, including age, gender and diagnosis. The diagnoses are reported to the service provider (e.g. from GPs, hospitals etc.) regarding the user and recorded in the service provider’s systems. Diagnoses are provided in ICPC-2 or ICD-10 (depending on the source). Diagnoses are only registered if considered relevant for the service provided.^24^

Data from primary health care practitioners (2) include individual level patient information on every visit to primary health care practitioners (including GPs, emergency room visits, physical therapists etc.). All diagnoses are provided in ICPC-2.

Importantly, while nursing home physicians *can* record diagnoses in NRPHC, only a small number of diagnoses are recorded and thus included in the registry.

By triangulating the data, we include all diagnoses given to all individuals using each long-term care institution, by any primary health care practitioner, as well as diagnoses provided from hospitals and private clinics if the diagnoses are reported to the service provider and recorded.

### Adverse Outcomes

We operationalize adverse outcomes as the proportion of registered service users with at least one new diagnosis each month within a set of predefined diagnoses (e.g., infections, accidents and injuries, depression, anxiety, malnutrition, dehydration). The categories and specific diagnoses are based on frequent adverse events and outcomes registered among institutionalized elderly patients,^25^ and diagnoses indicating quality of care in prior studies.^26,27^ Table 2 lists the frequency of all included diagnoses. Appendix C includes a detailed description of each code included from ICPC-2 or ICD-10.

For each individual user, we count if they receive at least one of these new diagnoses each month from 2018 to 2022. The data is then aggregated to the organizational level. Each organization is given a value each year from 0 to 100 representing the average proportion of their users with an adverse outcome each month that year. Because the shift schedule is reported on 1 May each year, the yearly average is calculated from 1 May of the survey year to 30 April of the following year.

#### Population: Organizations and Service Users

For the purpose of this study, we restrict the data to organizations that have a compressed work schedule and/or two or three-shift rotations. We exclude organizations that had implemented a compressed schedule the first year they appear in the data because they lack pre-treatment information. For organizations that stop using a compressed schedule, we exclude every year they no longer have a compressed schedule. We also exclude organizations with 24-hour shift rotations. Thus, at baseline, all organizations have a two-shift rotation (some employees rotate between day and evening shifts, others work fixed night shifts) and/or a three-shift rotation (same employees rotating across day, evening and night shifts). We then follow them as some implement a compressed schedule while others do not.

Finally, we also limit our study to service users that have received services for more than one month and exclude organizations without registered users from the Norwegian Registry for Primary Health Care (NRPHC) that meet this criteria. Some elderly people are sent to nursing homes for care in the very last days of their lives, and in these cases, it is less likely that any diagnoses or adverse event should be attributed to the quality of care in the institution.

In cases where users receive services from more than one organization in our dataset during the same month, the user is sorted within the organization where they receive the most comprehensive care and the care they have received the longest prior to that month.

Based on these criteria, the final analytical sample comprised 1,031 organizations, each with data from on average 100 service users per month. In total, 320 of these organizations implemented a compressed schedule during the study period.

#### Analyses

We use a difference-in-differences method to analyze how the proportion of service users with adverse outcomes change over time in organizations that implement compressed schedules compared to those that do not.

The intervention group comprised organizations operating a two- and/or three-shift rotation in 2018 (or at their first appearance in the dataset) that introduced a compressed schedule between 2019 and 2022. The control group included organizations that maintained a two- and/or three-shift rotation (i.e. never treated). The first intervention year was defined as the 12 months between an organization’s survey response indicating no compressed schedule per 1 May of one year and its response indicating a compressed schedule per 1 May of the following year (i.e. from first of May of the previous year through April of the current year)(Ideally the time span should have been 2 of May until 1 of May – however as dates in NRPHC were given in year and month (not precise dates) due to anonymity concerns – this was not possible.).

Because organizations implemented a compressed schedule at different time points during the period we use Callaway and Sant’Anna’s approach to Difference-in-differences (DiD) analysis with multiple treatment times^28^ and the csdid module in Stata 17.^29^ The csdid estimator is more resilient to violations of the parallel trends assumption and to imbalanced panels (i.e. where the number of observations is not the same for all entities) than jwdid because it uses group-time-specific treatment effects instead of pooling information across time and groups.

To combat challenges with any breach of parallel trends assumptions we ran the analyses with doubly robust inverse probability weighting (dripw) and control variables, including type of institution (nursing home, home care services, or residential care organizations), and the number and average age of service users. The control variables were assumed to be exogenous and unaffected by the treatment and were therefore included both pre and post treatment.

We first conducted a main analysis including all organizations and all types of compressed work schedules. To test the robustness of the findings, we performed a series of stratified analyses. The sample was stratified by type of compressed shift (extended shifts throughout the week or only on weekends) and by type of organization (nursing homes, residential care facilities, and home care services). We then conducted three additional analyses for all organization types, restricting the intervention group to those where (1) more than 60% of the nurses worked a compressed schedule, (2) more than 60% of health care assistants worked a compressed schedule, or (3) more than 60% of at least one employee group worked a compressed schedule. To examine whether combining multiple diagnoses might mask effects on individual conditions, we ran separate analyses for each diagnosis across all organizations.

Finally, to assess whether any treatment effect on the composite diagnosis outcome was *practically null*, we conducted equivalence testing around zero using the two one-sided tests (TOST) procedure.^30^ We specified equivalence bounds [−Δ,+Δ] centered at 0 and concluded equivalence if the entire 90%(90% Cl (1 − 2α) is used instead of a 95% CI (1 − α) since two one-sided tests (each with an α of 5%) are performed, for a discussion see Lankens (2017).) confidence interval for the DiD estimate lay within these bounds.^31^ We used two approaches to determine the equivalence bounds (smallest effect size of interest, SESOI): one anchored in mortality and one based on Cohens’d. An independent researcher set both bounds a priori, before seeing the results.

For the first SESOI we anchored the limit at 1% predicted annual change in mortality. Changes below this are considered practically null. To keep the estimate conservative, we assume a mortality rate of 25% for all diagnoses, the expected mortality rate for pneumonia. Based on typical pneumonia case-fatality rates in similar populations ranging from 23%^32^ to 28.7 %,^33^ pneumonia is one of the most severe diagnoses in our study. This estimation yielded equivalence bounds of ±0.32 percentage points per month for the composite outcome (i.e. ((1% yearly change/25% mortality)/12month)).

For the second SESOI we used a standardized approached. Lakens, Scheel and Isager^34^ describes the option of using a subjective measure, which we set to Cohen’s *d* = 0.1, (trivial effect size) corresponding to ±0.73 percentage points. Cohen’s d expresses the size of difference in SD independent of sample size. While d=0.2 is conventionally considered a small effect size,^35^ we adopted a stricter threshold of d=0.1, often classified as very small or negligible.^35^ Our reasons for choosing this more restrictive boundary were that in the context of patient safety and adverse outcomes, even a conventionally small increase in harm (d=0.2) could be considered problematic, especially since we do not know which diagnoses change in our composite measure.

#### Patient and Public Involvement

The current project is executed in collaboration with the three largest unions representing employees in long-term care as well as one Norwegian municipality. The collaborating partners have provided feedback and been valuable sparring partners for the research questions, development of the organizational survey, and interpretation of the results and the context they occur in. As key stakeholders in the decision to implement compressed schedules they are also important for securing the dissemination and use of the findings.

## Results

The analytical sample is presented in table 1. The data consist of 1,031 organizations and 4,869 observations (organization-years). A total of 320, or 31%, of the included organizations implemented a compressed schedule between May 2018 and May 2022. Between 71 and 89 organizations implemented a compressed schedule each year – a steady increase for each year of the study. Implementing a compressed schedule was more common among residential care organizations (39%) than nursing homes (28%) and home care (25%). The majority of units implemented a compressed schedule where employees only worked extended shifts during weekends (72% of all organizations with a compressed schedule). Fewer organizations implemented a compressed schedule in which employees could work extended shifts on any weekday (21%). In these cases, most employees still worked a combination of extended and shorter shifts.

**Table 1.** Number (N) of Organizations and Observations Across Organizations

​	Organizations	Observations (organization years)
Total N	1031	4869
Never treated	711	3368
Any Compressed work schedule (CWS, treated)	320	1501
CWS-Cohort year 2018/2019	71	336
CWS-Cohort year 2019/2020	78	351
CWS-Cohort year 2020/2021	82	385
CWS-Cohort year 2021/2022	89	429
CWS weekends	229	1048
CWS all week	68	307
CWS other/missing	23	​
Nursing homes	357	1717
Never treated	256	1244
Any CWS (treated)	101	473
Residential care	369	1717
Never treated	225	1035
Any CWS (treated)	144	682
Home care services	305	1435
Never treated	230	1089
Any CWS (treated)	75	346

Table 2 presents the descriptive statistics for the included organizations. Each organization has on average 100 service users with a mean age of 71. Overall, 13% of the users were diagnosed with at least one new diagnosis each month, the most common diagnoses were infections (3.6%), accidents (including falls, accidents and injuries to users, 2.5%), depression (1.3%), and sleep disorders (1.1 %). Residential care had the fewest users per organization and the youngest user group, reflecting that it serves not only older adults but also individuals with physical and mental disabilities and substance abuse. Home care had more users per organization and the highest percentage of new diagnoses per service user. Nursing homes, by contrast, had the oldest user group and the lowest percentage of new diagnoses per user.

**Table 2.** Service Users and Adverse Outcomes Across Organizations

​	Total		Nursing homes		Residential care		Home care	
	mean	sd	mean	sd	mean	sd	mean	sd
Number of service users per month	100	154	82	77	21	38	215	225
Mean age of service users	71	18	85	5	55	18	73	11
Adverse outcomes (proportion of service users with a new diagnosis per month)								
Any diagnosis	12.5 %	7.9	8.8 %	7.3	12.7 %	8.3	16.6 %	5.8
Anxiety	0.7 %	2.1	0.3 %	0.7	0.7 %	2.1	1.1 %	2.9
Anorexia/bulimia	0.0 %	0.2	0.0 %	0.1	0.0 %	0.3	0.0 %	0.1
Covid-19	0.5 %	1.5	0.4 %	1.3	0.7 %	1.9	0.5 %	1.0
Dehydration	0.2 %	0.4	0.3 %	0.5	0.1 %	0.4	0.2 %	0.2
Depression	1.3 %	2.5	0.6 %	1.1	1.2 %	2.6	2.3 %	3.2
Other/unspecified mood disorders/states	0.9 %	1.6	0.5 %	0.8	0.9 %	2.2	1.3 %	1.1
Falls, accidents and injuries	2.5 %	3.3	2.2 %	2.2	2.4 %	4.6	3.0 %	2.2
Malnutrition and other nutritional deficiencies	0.7 %	1.0	0.4 %	0.5	0.8 %	1.3	1.0 %	0.8
Weight loss/gain	0.1 %	0.3	0.1 %	0.2	0.1 %	0.5	0.2 %	0.3
Constipation	0.5 %	1.1	0.3 %	0.4	0.8 %	1.6	0.5 %	0.6
Infections	3.6 %	3.0	2.9 %	2.5	4.0 %	3.9	4.2 %	1.8
Influenza	0.1 %	0.2	0.0 %	0.1	0.1 %	0.3	0.1 %	0.2
Chronic skin ulcers (including bedsores/pressure ulcer)	0.5 %	0.9	0.3 %	0.5	0.5 %	1.3	0.8 %	0.8
Pneumonia	0.8 %	1.1	1.0 %	1.2	0.6 %	1.3	0.6 %	0.6
Dizziness	0.4 %	0.7	0.3 %	0.4	0.3 %	0.9	0.8 %	0.6
Sleep disorders	1.1 %	2.2	0.5 %	0.8	0.9 %	2.1	2.1 %	3.0
Fatigue	0.5 %	1.0	0.3 %	0.5	0.4 %	1.4	0.7 %	0.7

**Figure 1.**
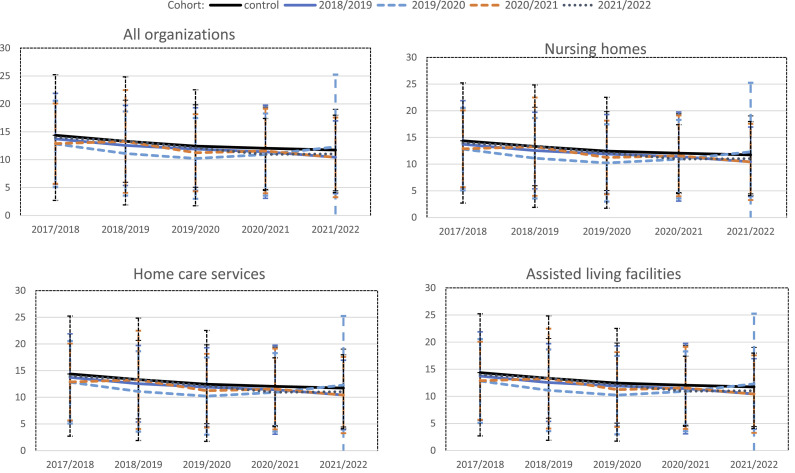
Percentage of adverse events per cohort and year *Note.* The figure shows adverse events each year (x-axis) per cohort, the bars represent 95% confidence intervals

Figure 1 illustrates the proportion of service users with an adverse event by year for each cohort (i.e. when they implemented a compressed schedule), in total and stratified by type of organisation.

Table 3 presents the results of the main difference-in-difference analyses, for all organizations and all types of compressed work schedule. The results show no significant change in adverse outcomes following the implementation of a compressed work schedule (ATT. -046, p=0.08 in adjusted model).

**Table 3.** Difference-In-Differences Analyses of Implementation of Compressed Work Schedules (Main Analysis)

​	Unadjusted		Adjusted	
	coef.	95% CI	coef.	95% CI
Average treatment effect on the treated (ATT)	-0.42	(-0.93 0.08)	-0.46	(-0.97 0.05)
3 years pre	-0.46	(-1.14 0.23)	-0.37	(-1.05 0.31)
2 years pre	-0.24	(-0.71 0.24)	-0.19	(-0.65 0.27)
1 year pre	-0.28	(-0.69 0.12)	-0.28	(-0.69 0.13)
Implementation	-0.13	(-0.44 0.18)	-0.14	(-0.45 0.17)
1 year post	-0.55	(-1.30 0.19)	-0.59	(-1.33 0.14)
2 years post	-0.86	(-1.85 0.13)	-0.93	(-1.95 0.09)
3 years post	-0.79	(-1.99 0.41)	-0.87	(-2.11 0.37)
Pre-trend test	​			
p-value	​	0.34	​	0.50

Looking at the development per year prior to and post implementing a compressed schedule we find no significant difference in development in any of the years prior to implementation (1 year pre -0.28 p= 0.19; 2 years pre -0.19 p= 0.41; and 3 years pre -0.37 p=0.28), the implementation year (-0.14 p=0.39), or any of the three years post intervention (1 year post -0.59 p=0.12; 2 years post -0.93 p=0.07; and 3 years post -0.87 p=0.17).

The pre-trend chi-square tests were non-significant (p-value = 0.5), indicating no significant differences in trends in the years prior to implementation and supporting the validity of the linear trends’ assumption.

**Figure 2.**
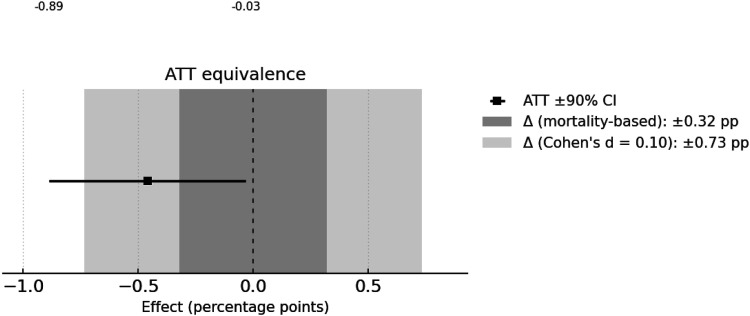
Equivalence test of difference in difference analyses for implementation of compressed work schedules (table 3)

Equivalence testing (Figure 2) of the main effect did not demonstrate practical equivalence to zero. Using the mortality-based bounds, Δ = ±0.32 pp, derived from pneumonia case-fatality rates, the 90% CI (−0.89 to−0.03 pp) for the treatment effect extended beyond the lower bound. The same pattern held for the standardized margin ±0.73 pp (Cohen’s *d* = 0.10), with the 90% CI not fully contained within the lower bounds. Consequently, we cannot determine if the main effect was negative or equivalent to zero. This result, however, appears contingent on the use of strict bounds, as the confidence interval lies within the range typically classified as a small effect size (Cohen’s *d* = 0.2^35^).

To test the robustness of the findings we have replicated the main analyses stratified by type of compressed shift (table 4), type of organization (Table 5), limited to organizations where more than 60% of employee groups work a compressed schedule (Table 6), and for each individual diagnosis (Table 7). Table 4 shows no significant effects when implementing a compressed schedule with extended shifts throughout the week, or when implementing a compressed schedule with extended shifts only on weekends. Table 5 shows no significant effect when implementing a compressed schedule in residential care or home help. However, the results did show a significant reduction in adverse outcomes when implementing a compressed schedule in nursing homes.

**Table 4.** Difference-In-Differences Analyses Stratified by Type of Compressed Schedule

​	Throughout week		Only weekends	
	coef.	95% CI	coef.	95% CI
ATT	-1.39	(-2.98 0.20)	-0.22	(-0.68 0.24)
3 years pre	-0.83	(-2.28 0.63)	-0.32	(-1.05 0.42)
2 years pre	0.89	(-0.27 2.06)	-0.34	(-0.85 0.16)
1 year pre	-0.64	(-1.77 0.49)	-0.29	(-0.75 0.16)
Implementation	-0.53	(-1.47 0.41)	0.02	(-0.32 0.36)
1 year post	-1.37	(-3.63 0.89)	-0.37	(-1.09 0.34)
2 years post	-3.38	(-7.45 0.69)	-0.52	(-1.40 0.36)
3 years post	-6.35**	(-11.10 1.61)	-0.62	(-2.03 0.79)
Pre-trend test	​			
p-value	​	0.10	​	0.38

*p < 0.05; **p < 0.01; ***p < 0.001

**Table 5.** Difference-in-Differences Analyses Stratified by Type of Organization

​	Nursing homes		Residential care		Home care	
	coef.	95% CI	coef.	95% CI	coef.	95% CI
ATT	-0.79*	(-1.48 -0.10)	-0.20	(-1.11 0.70)	-0.42	(-1.20 0.36)
3 years pre	-0.04	(-1.08 1.01)	-0.34	(-2.58 1.91)	-0.52	(-1.52 0.48)
2 years pre	0.13	(-0.56 0.81)	0.72	(-0.53 1.97)	-0.47	(-1.36 0.42)
1 year pre	-0.45	(-1.14 0.25)	-1.41*	(-2.52 -0.31)	-0.07	(-0.68 0.54)
Implementation	-0.39	(-0.80 0.03)	-0.13	(-1.12 0.85)	-0.04	(-0.53 0.44)
1 year post	-0.86	(-1.72 0.01)	-0.50	(-1.68 0.69)	-0.55	(-1.71 0.61)
2 years post	-0.97	(-2.20 0.26)	0.08	(-1.13 1.29)	-1.45	(-3.15 0.26)
3 years post	-2.26*	(-4.17 -0.36)	-0.24	(-1.79 1.31)	-0.45	(-2.40 1.50)
Pre-trend test	​					
p-value	​	0.82	​	0.20	​	0.78

*p < 0.05; **p < 0.01; ***p < 0.001

**Table 6.** Difference-in-Differences Analyses Limited to Organizations Where More Than 60% Work Compressed

​	More than 60% of nurses		More than 60% of health care assistants		More than 60% of at least one employee group	
	coef.	95% CI	coef.	95% CI	coef.	95% CI
ATT	-0.08	(-0.73 0.58)	0.08	(-0.69 0.85)	-0.34	(-0.95 0.27)
3 years pre	0.01	(-0.83 0.85)	-0.29	(-1.39 0.81)	-0.18	(-0.98 0.62)
2 years pre	-0.17	(-1.27 0.93)	0.14	(-1.32 1.60)	-0.11	(-1.02 0.80)
1 year pre	-0.38	(-1.17 0.42)	-0.23	(-1.34 0.89)	-0.34	(-1.04 0.37)
Implementation	0.14	(-0.48 0.77)	0.35	(-0.41 1.11)	-0.04	(-0.56 0.49)
1 year post	-0.09	(-1.43 1.25)	-0.16	(-1.89 1.58)	-0.40	(-1.52 0.72)
2 years post	-0.32	(-1.20 0.56)	0.14	(-0.92 1.21)	-0.52	(-1.43 0.38)
3 years post	-0.80	(-1.64 0.04)	-0.73	(-1.56 0.10)	-1.38*	(-2.56 -0.21)
Pre-trend test	​					
p-value	​	0.75	​	0.37	​	0.83

*p < 0.05; **p < 0.01; ***p < 0.001

**Table 7.** Difference-In-differences Analyses by Diagnosis

​	ATT	P	95% CI
Anxiety	-0.03	​	(-0.21 0.15)
Anorexia/bulimia	-0.01	​	(-0.03 0.01)
Covid-19	-0.12	​	(-0.30 0.07)
Dehydration	-0.05	*	(-0.09 0.00)
Depression	-0.21	​	(-0.43 0.02)
Other/unspecified mood disorders/states	-0.04	​	(-0.17 0.10)
Falls, accidents and injuries	0.02	​	(-0.22 0.27)
Malnutrition and other nutritional deficiencies	-0.06	​	(-0.20 0.07)
Weight loss/gain	-0.01	​	(-0.05 0.03)
Constipation	0.01	​	(-0.08 0.10)
Infections	0.12	​	(-0.19 0.43)
Influenza	-0.02	​	(-0.05 0.02)
Chronic skin ulcer	0.04	​	(-0.10 0.17)
Pneumonia	-0.04	​	(-0.11 0.03)
Dizziness	-0.10	*	(-0.20 0.00)
Sleep disorders	0.18	​	(-0.02 0.37)
Fatigue	0.05	​	(-0.03 0.13)

*p < 0.05; **p < 0.01; ***p < 0.001

Table 6 shows no significant change when implementing a compressed schedule where more than 60% of registered nurses in the organization work extended shifts, more than 60% of licensed practical nurses work extended shifts or more than 60% of at least one employee group work extended shifts. Finally, Table 7 shows no significant change in 15 of the 17 included diagnoses, while two (dehydration and dizziness) show a significant decrease in frequency following the implementation of compressed schedules.

## Discussion

This study found no overall effect of compressed work schedules on adverse outcomes in long-term care institutions. The analyses, including equivalence testing, indicate that extended shifts within compressed schedules can be implemented with on average little or no increase in adverse diagnostic outcomes.

Furthermore, no significant detrimental effects were observed in any of the subgroups investigated, while a significant reduction in adverse outcomes was identified in nursing homes. The observed positive association in nursing homes should be interpreted with caution, as the use of multiple stratified analyses increases the risk of type I error. Nonetheless, the positive relationship in nursing homes indicates that effects of a compressed schedule may be context specific, depending on the type of institution. Potential benefits of compressed schedules, such as reduced handovers, may be particularly salient in nursing home settings, where staff maintain close and continuous contact with residents.

Several studies have reported detrimental effects of extended shifts on performance and patient safety.^1-5^ However, these findings largely stem from settings where extended shifts are not organized within a compressed schedule. The limited number of studies specifically examining compressed shift work have generally found no difference, or even improvements, in patient outcomes.^3,8,9^ The present study adds to this emerging evidence, by finding no significant increase in risk of adverse diagnostic outcomes among service users. Moreover, the significant reduction in nursing homes indicate that differences between institutions and context is one potential reason for the mixed findings in prior studies. Importantly, compared with previous studies, this study draws on a substantially larger sample and a longer observation period, thereby providing stronger statistical power and greater robustness to the null findings. Moreover, to our knowledge, this is the first study to evaluate the effects of compressed schedules on service users in long-term care settings.

### Strengths and Limitations of the Study

A major strength of this study is the use of a nationwide organizational survey covering 1,031 institutions over a five-year period. Although the schedule data were collected retrospectively, the introduction of a compressed schedule typically involves a formal application and implementation process, making it likely that employers accurately recall the timing of adoption.

The nationwide sample strengthens the generalizability of the findings across multiple organizations. However, the sample consists primarily of publicly owned organizations. All implementations occurred within Norwegian legislation, including the Norwegian work hour restrictions and mandatory approval from unions for the implementation of compressed work schedules. A different context could yield different results. Similarly, the COVID-19 pandemic started during the study period. The pandemic may have impacted organizations’ motives for a compressed schedule and outcomes (reducing infection by reducing number of staff per day). The results are, however, relatively stable across cohorts and diagnoses.

Analyzing outcomes at the organizational level represents an additional strength, as it captures the combined influence of individual- and organization-level mechanisms. Such mechanisms may work in opposite directions. For example, extended shifts may increase fatigue and the risk of error, whereas fewer handovers may reduce this risk and enhance continuity of care. Assessing the net effect therefore provides valuable insight into the overall consequences for service users. At the same time, the absence of an overall effect may conceal opposing mechanisms operating simultaneously.

A limitation of the current study is that we do not know when the implementation occurred, only which year. Consequently, all data is aggregated to years. During the implementation year, organizations may have had a compressed schedule for 1 day only or 364. This may lead to an underestimation of the effect during the implementation year, which could be indicated by a lower coefficient during the implementation year than the following year. However, neither coefficient is significant.

For the current study we performed no sample size or power analysis to determine the size of the population. The survey was sent to all existing public long-term care institutions in Norway. While the current sample is substantial in terms of both number of organizations and users, it is also important to note that absence of evidence is not evidence of absence.^36^ For the main analysis we address this limitation by including non-inferiority tests.

Finally, an important strength and limitation is the use of diagnostic records to capture adverse outcomes among service users. The data provides a nation-wide longitudinal recording of patient outcomes assessed by a physician. However, several limitations with the use of these data is important to acknowledge.

First, not all diagnoses are included in our data. Data from the NRPHC consists of diagnoses reported from primary health-care practitioners and the municipal healthcare services. Diagnoses from specialist healthcare institutions (e.g., hospitals) are only recorded in the NRPHC if they are reported to the municipality and considered relevant for the service provided by the municipality. How often diagnoses from specialist healthcare are recorded in NRPHC varies by diagnosis. One study found that NRPHC included 88% diagnoses for “other mental disorders” recorded in either NRPHC or NPR (registry for specialist healthcare), but only 53% of hip fractures.^24^ Second, some diagnoses or health concerns, such as dementia, are often not recorded in any registry.^24^ In our data underdiagnosis appears particularly common in nursing homes. One explanation is that physicians in nursing homes may lack incentives for recording diagnoses, as their patients already receive the highest level of care.^24^ In contrast, GPs who provide medical care in home-based services are required to record diagnoses and use diagnoses to show degradation of health for nursing home applications. Future studies should aim to incorporate additional registries such as registries for mortality and specialist healthcare.

Third, several dimensions of care quality may not be reflected by diagnoses. Quality of care is often measured as outcome (as in the current paper), process or structure.^37^ Compressed schedules could also impact structure (e.g. increased qualifications of staff if a more attractive schedule improves recruitment or retention) or process (e.g. if increased flexibility afforded by the extended shifts impacts tasks left undone, activities during the day, or the use of force or medication because employees can adjust the timing of activities to when employees are willing). Moreover, a compressed schedule may also affect other outcomes than diagnoses, such as users’ experiences of stress, meaningfulness and quality of life.

### Implications for Policymakers

In this study, we found no evidence of an overall change in adverse outcomes among service users following the introduction of compressed schedules. The finding has two important implications for policy makers. For one, while we cannot exclude a positive relationship, the current study yields limited support for the notion that compressed schedules should be implemented to improve quality of care. However, the results also suggest that policymakers and service managers may consider implementing compressed schedules in long-term care for workforce or organizational reasons without (on average) compromising care quality. Compressed schedules are implemented for a variety of reasons, including improving employees’ work-life balance^8^ and making more efficient use of staffing resources.^38^ Several studies have also supported that employees working a compressed schedule are more satisfied with their own shift arrangements.^9^ In Norway, compressed schedules have often been implemented as a tool to address staffing shortages or due to employee preferences. For practitioners considering implementing compressed schedules to elicit these benefits, the current study indicates that they may do so without increasing adverse events.

### Conclusion

This study found no overall change in adverse outcomes following the introduction of compressed schedules, while indicating potential context-specific benefits. The findings support that extended shifts within a compressed schedule can be implemented in long-term care services without on average increasing adverse outcomes.

## Supplemental Material

Supplemental Material - Do Compressed Work Schedules Compromise Care Quality? A Five-Year Case Control Study in the Norwegian Long-Term Care Sector Supplemental Material for Do Compressed Work Schedules Compromise Care Quality? A Five-Year Case Control Study in the Norwegian Long-Term Care Sector by Vilde Hoff Bernstrøm, Mari Ingelsrud, Karoline Seglem, Anne-Marthe Rustad Indregard in Health Services Insights

Supplemental Material - Do Compressed Work Schedules Compromise Care Quality? A Five-Year Case Control Study in the Norwegian Long-Term Care Sector Supplemental Material for Do Compressed Work Schedules Compromise Care Quality? A Five-Year Case Control Study in the Norwegian Long-Term Care Sector by Vilde Hoff Bernstrøm, Mari Ingelsrud, Karoline Seglem, Anne-Marthe Rustad Indregard in Health Services Insights

Supplemental Material - Do Compressed Work Schedules Compromise Care Quality? A Five-Year Case Control Study in the Norwegian Long-Term Care Sector Supplemental Material for Do Compressed Work Schedules Compromise Care Quality? A Five-Year Case Control Study in the Norwegian Long-Term Care Sector by Vilde Hoff Bernstrøm, Mari Ingelsrud, Karoline Seglem, Anne-Marthe Rustad Indregard in Health Services Insights

## Data Availability

The current paper utilizes two set of data. The registry data may be applied for through the platform https://helsedata.no/en/. An anonymous version of the organizational survey will be made openly available and provided by the first author upon reasonable request.
