# Supplementary material for: Do Compressed Work Schedules Compromise Care Quality? A Five-Year Case Control Study in the Norwegian Long-Term Care Sector
Source: Health Serv Insights. 2026 Aug 18;19:11786329261471203. doi: 10.1177/11786329261471203 (PMC13487131; doi:10.1177/11786329261471203)
Supplement: Supplementary file 1 [file sj-pdf-1-his-10.1177_11786329261471203.pdf]

Appendix A: Response rate in detail

		Number of units	Number of responses	Response rate
Type of institution	Nursing home	907	389	43 %
	Home care	1017	374	37 %
	Residential care	2254	651	29 %
Ownership	Private	388	4	1 %
	Municipal	3793	1439	38 %
Municipality size	Small (< units)	2052	816	40 %
	Medium	969	387	40 %
	Large	1156	237	21 %
Organization size	Small (< 31 employees)	1378	270	20 %
	Medium (31–60)	1335	491	37 %
	Large (61+)	1464	679	46 %
Total		4181	1443	35 %

The table is translated from Bernstrøm VH, Fevang E, Gautun H, Ingelsrud MHL, Andreas, Lillebø OS. Dokumentasjon av data fra undersøkelse om turnusordninger i kommunenes helse- og omsorgstjenester. Oslo: Frischenteret; 2022.
