# Supplementary material for: Do Compressed Work Schedules Compromise Care Quality? A Five-Year Case Control Study in the Norwegian Long-Term Care Sector
Source: Health Serv Insights. 2026 Aug 18;19:11786329261471203. doi: 10.1177/11786329261471203 (PMC13487131; doi:10.1177/11786329261471203)
Supplement: Supplementary file 2 [file sj-pdf-2-his-10.1177_11786329261471203.pdf]

Appendix B Suvery question.

Shift types

Source: Adapted from the Shift survey. Barton, J., Spelten, E., Totterdell, P., Smith, L., Folkard, S., & Costa, G. (1995). The Standard Shiftwork Index: a battery of questionnaires for assessing shiftwork-related problems. Work & Stress, 9(1), 4-30.

The first question is about how the organization has structured the workday. We are asking about different types of shift schedules:

- **2-shift rotation**: The day is divided into three parts, where some employees rotate between working day and evening shifts, while others work permanent night shifts.
- **3-shift rotation**: The day is divided into three parts, and the same employees work day, evening, and night shifts.
- **Compressed work schedules**: Extended shifts lasting more than 10 hours that are part of the employees' planned schedule, where employees work longer but fewer shifts. Extended shifts can be used throughout the week or only at weekends. This does not include prolonged shifts that arise due to special needs or employees occasionally working double shifts. Nor does it include live-in shifts (see below).
- **24-hour shifts/live-in shifts**: A schedule where employees are at work for 24 hours or more, and the shift includes both active work and on-call/resting time.

3) Which of the following shift solutions has your organization had over the last four years? (Multiple answers possible. Tick the years your organization had each of the different shift solutions, even if only a small group of employees worked that type of shift.) Please provide the status as of May 1 each year.

	2-shift rotation	3-shift rotation	Compressed work schedules	24-hour shifts	Other	Don’t know
May 1, 2022						
May 1, 2021						
May 1, 2020						
May 1, 2019						
May 1, 2018						

Weekends or all weekdays

We would now like to know whether employees work extended shifts on all weekdays or only in connection with weekend shifts (e.g., by working extended shifts on Saturday and Sunday).

When did employees work extended shifts during a workweek? (Multiple answers possible). Please provide the status as of 1st May each year.

	All weekdays	Only weekends	Other	Don’t know
1 May 2022				
1 May 2021				
1 May 2020				
1 May 2019				
1 May 2018				
