# Supplementary material for: Do Compressed Work Schedules Compromise Care Quality? A Five-Year Case Control Study in the Norwegian Long-Term Care Sector
Source: Health Serv Insights. 2026 Aug 18;19:11786329261471203. doi: 10.1177/11786329261471203 (PMC13487131; doi:10.1177/11786329261471203)
Supplement: Supplementary file 3 [file sj-pdf-3-his-10.1177_11786329261471203.pdf]

Appendix C adverse outcomes and diagnoses

Adverce outcome	Table	Code	Name
anorexia/bulimia	ICD-10	F500	Anorexia nervosa
		F501	Atypical anorexia nervosa
		F502-F504	Bulimia
	ICPC-2	P86	Anorexia nervosa/bulimia
anxiety	ICD-10	F41	Other anxiety disorders
	ICPC-2	P01	Feeling anxious/nervous/tense
		P74	Anxiety disorder/anxiety state
chronic skin ulcer (including bedsores/pressure ulcer)	ICD-10	L89	Osteoarthrosis of hip
	ICPC-2	S97	Chronic ulcer skin
constipation	ICD-10	K564	Other impaction of intestine
		K590	Constipation
	ICPC-2	D12	Constipation
covid-19	ICD-10	U071	COVID-19
		U072	COVID-19, virus not identified
	ICPC-2	R991	Covid-19 Suspicion
		R992	Covid-19 confirmed
dehydration	ICD-10	E86	Volume depletion
	ICPC-2	T11	Dehydration
depression	ICD-10	F32	Depressive episode
		F33	Recurrent depressive disorder
		F341	Dysthymic disorder
	ICPC-2	P03	Feeling depressed
		P76	Depressive disorder
dizziness	ICD-10	R42	Dizziness and giddiness
	ICPC-2	N17	Vertigo/dizziness
falls, accidents and injurys	ICD-10	S00-T14	Injuries
		T20-T32	Burns and corrosions
		T33-T35	Frostbite
		T4n-T65	Poisoning
		T67	Effects of heat and light
		T68	Hypothermia
		V0n-V99	Transport accidents
		W0n	Falls (Acidents)
		W2s-X59	Other external causes of accidental injury
	ICPC-2	A80	Trauma/injury NOS
		A81	Multiple trauma/injuries
		A82	Secondary effect of trauma
		A84	Poisoning by medical agent
		A86	Toxic effect non-medicinal substance
		A88	Adverse effect physical factor
		B76	Ruptured spleen traumatic
		D79	Foreign body digestive system
		D80	Injury digestive system other
		F75	Contusion/haemorrhage eye
		F76	Foreign body in eye
		F79	Injury eye other
		H76	Foreign body in ear
		H78	Superficial injury of ear

		H79	Ear injury other
		L72	Fracture: radius/ulna
		L73	Fracture: tibia/fibula
		L74	Fracture: hand/foot bone
		L75	Fracture: femur
		L76	Fracture: other
		L77	Sprain/strain of ankle
		L78	Sprain/strain of knee
		L79	Sprain/strain of joint NOS
		L80	Dislocation/subluxation
		L81	Injury musculoskeletal NOS
		L96	Acute internal damage knee
		N79	Concussion
		N80	Head injury other
		N81	Injury nervous system other
		R87	Foreign body nose/larynx/bronchus
		R88	Injury respiratory other
		S11	Skin infection post-traumatic
		S14	Burn/scald
		S16	Bruise/contusion
		S18	Laceration/cut
		S19	Skin injury other
		U80	Injury urinary tract
		X82	Injury genital female
		Y80	Injury male genital
fatigue	ICD-10	G933	Postviral fatigue syndrome
		R53	Malaise and fatigue
	ICPC-2	A04	Weakness/tiredness general
infections	ICD-10	A419	Sepsis, unspecified organism
		J00-J06	Acute upper respiratory infections
		J20-J22	Other acute lower respiratory infections
		J40	Bronchitis, not specified as acute or chronic
		L00-L08	Infections of the skin and subcutaneous tissue
		N30	Cystitis
		N390	Urinary tract infection, site not specified
	ICPC-2	A78	Infectious disease other/NOS
		B70	Lymphadenitis acute
		R72	Strep throat
		R74	Upper respiratory infection acute
		R75	Sinusitis acute/chronic
		R76	Tonsillitis acute
		R77	Laryngitis/tracheitis acute
		R78	Acute bronchitis/bronchiolitis
		R83	Respiratory infection other
		S09	Infected finger/toe
		S10	Boil/carbuncle
		S76	Skin infection other
		S84	Impetigo
		S85	Pilonidal cyst/fistula

		U71	Cystitis/urinary infection other
influenza	ICD-10	J09-J11	Influenza
	ICPC-2	R80	Influenza
malnutrition and other nutriononal deficiencies	ICD-10	E40-E46	Malnutrition
		E50-E64	Other nutritional deficiencies
		R636	Underweight
		T730	Effects of hunger
	ICPC-2	T91	Vitamin/nutritional deficiency
mental disorders due to known physiological conditions (other than dementia)	ICD-10	F04	Visual floaters/spots
		F05	Visual disturbance other
		F06	Other mental disorders due to brain damage and dysfunction and to physical disease
		F07	Personality and behavioural disorders due to brain disease, damage and dysfunction
		F09	Unsp mental disorder due to known physiological condition
	ICPC-2	P71	Organic psychosis other
other/unspecified mood disorders/states	ICD-10	F348-F349	other/unspecified mood disorders
		F38	Other mood [affective] disorders
		F39	Unspecified mood [affective] disorder
		R45	Symptoms and signs involving emotional state
	ICPC-2	P01	Feeling anxious/nervous/tense
		P02	Acute stress reaction
		P04	Feeling/behaving irritable/angry
pneumonia	ICD-10	J12-J18	Pneumonia
	ICPC-2	R81	Pneumonia
sleep disorders	ICD-10	F510	Nonorganic insomnia
		F511	Nonorganic hypersomnia
		F512-F519	Other Sleep disorders
		G470	Disorders of initiating and maintaining sleep [insomnias]
		G471	Disorders of excessive somnolence [hypersomnias]
		G472-G479	Sleep disorders
	ICPC-2	P06	Sleep disturbance
weight loss/gain	ICD-10	R634	Abnormal weight loss
		R635	Abnormal weight gain
		R64	Cachexia
	ICPC-2	T07	Weight gain
		T08	Weight loss
